# Response of *Microcystis aeruginosa* and Microcystin-LR to electron beam irradiation doses

**DOI:** 10.1016/j.radphyschem.2021.109534

**Published:** 2021-04-28

**Authors:** Alexandra M. Folcik, Cory Klemashevich, Suresh D. Pillai

**Affiliations:** a Interdisciplinary Graduate Program in Toxicology, Texas A&M University, College Station, TX, USA; b National Center for Electron Beam Research, An IAEA Collaborating Centre for Electron Beam Technology, Texas A&M University, College Station, TX, USA; c Integrated Metabolomic Analysis Core, Texas A&M University, College Station, TX, USA

**Keywords:** Harmful algal blooms, Microcystin, Cyanobacteria, Ionizing radiation, Water

## Abstract

Harmful cyanobacterial blooms (cyanoHABs) pose threats to human and animal health due to the production of harmful cyanotoxins. *Microcystis aeruginosa* is a common cyanobacterium associated with these blooms and is responsible for producing the potent cyclic hepatotoxin microcystin-LR (MC-LR). Concerns over the public health implications of these toxins in water supplies have increased due to rising occurrence of these blooms. High energy electron beam (eBeam) irradiation technology presents a promising strategy for the mitigation of both cyanobacterial cells and cyanotoxins within the water treatment process. However, it is imperative that both cellular and chemical responses to eBeam irradiation are understood to ensure efficient treatment. We sought to investigate the effect of eBeam irradiation on *M. aeruginosa* cells and MC-LR degradation. Results indicate that doses as low as 2 kGy are lethal to *M. aeruginosa* cells and induce cell lysis. Even lower doses are required for degradation of the parent MC-LR toxin. However, it was observed that there is a delay in cell lysis after irradiation where *M. aeruginosa* cells may still be metabolically active and able to synthesize microcystin. These results suggest that eBeam may be suitable for cyanoHAB mitigation in water treatment if employed following cell lysis.

## 1. Introduction

Harmful algal blooms (HABs) are increasing in occurrence worldwide due to warming water temperatures and increasing anthropogenic nutrient pollution (Paerl and Otten, 2013). Many of these blooms are associated with the production of various hepato- and neuro-toxins, as well as odor and taste compounds. HABs can occur in freshwater, brackish water, and in marine environments. However, when these blooms occur in freshwater, they are in fact often caused by cyanobacteria, and not algae (cyanoHABs). An increased threat to public health arises when these cyanoHABs occur in lakes, reservoirs, and other source waters which can then be taken into water treatment plants (Sharma et al., 2012). This occurred in Toledo, OH in 2014 when a bloom in Lake Erie caused the shut off of public drinking water due to cyanotoxin contamination (Steffen et al., 2017). There is increasing concern within the water industry about the occurrence of cyanoHABs in source water and of the toxins passing through drinking water treatment plants (Pelaez et al., 2010).

*Microcystis aeruginosa* is among the most common cyanobacteria in cyanoHABs and is the main species responsible for producing microcystin, a potent liver toxin (Tanabe et al., 2018). Of the over 200+ identified variants, microcystin-LR (MC-LR) is the most common and most toxic (Buratti et al., 2017; Munoz et al., 2019). The 3-amino-9-methoxy-2,6,8-trimethyl-10-phenyldeca-4,6-dienoic acid (ADDA) side group of the molecule is thought to be largely responsible for its toxicity (Valério et al., 2010). The toxin acts by irreversibly binding and inhibiting the liver enzymes protein phosphatase 1 (PP1) and protein phosphatase 2A (PP2A) as well as activating phosphorylase B enzymes. This in turn results in apoptosis of hepatocytes caused by a sequence of events leading to excessive phosphorylation of cytoskeletal filaments (Valério et al., 2010).

Treatment technologies are needed to address not only these cyanotoxins, but also the toxin-producing cyanobacterial cells. Conventional treatments such as coagulation, flocculation, sedimentation, and filtration are generally effective at removing cyanobacterial cells, given that the cells remain intact (Chow et al., 1999; Newcombe and Nicholson, 2004). Still, during extensive blooms, conventional treatments may not be enough to remove the abundance of cells. What is more, these conventional treatments result in residual products like sludges and sediments that contain the hazardous organisms and may require further treatment (Maghsoudi et al., 2015; Ranković et al., 2020; Xu et al., 2016).

However, if the *M. aeruginosa* cells are lysed, MC-LR is released into the water, and if not addressed may intrude into the drinking water distribution system (Fan et al., 2014). Chemical oxidation involving chlorine, potassium permanganate, copper sulfate, and hydrogen peroxide have been found largely ineffective and all have the additional issue of generating harmful disinfection byproducts (Sharma et al., 2012). Other advanced oxidation processes (AOPs) have been studied for MC-LR removal including UV/H_2_O_2_, Fenton reagent, modified natural magnetite, and ozone, with many of the AOPs being able to degrade MC-LR at ideal conditions (Chang et al., 2014; Munoz et al., 2019; Park et al., 2017; Zong et al., 2013). However, presently drinking water treatment plants utilize ozonation. Though ozonation is effective at breaking down the MC-LR molecules, studies show that ozone’s effectiveness is highly dependent on stringent operational requirements including low dissolved organic content, optimal pH, and optimal temperatures (Rositano et al., 2001; Teixeira and Rosa, 2007). Moreover, ozone has been shown to lyse *M. aeruginosa* cells causing a release of intracellular microcystin. As a result, the US EPA has advised against pre-treatment oxidation of drinking water to prevent this cell lysis and the release of microcystins into the distribution system (United States Environmental Protection Agency, 2014). Therefore, a technology is needed that not only has the capacity to inactivate the cyanobacterial cells but that is also capable of degrading the toxin molecule. This will ensure that irrespective of where the technology is deployed, the risks of toxin intrusion into the distribution system is eliminated.

High energy electron beam (eBeam) technology is an advanced oxidation-reduction process (AORP) that has been demonstrated to be effective against a variety of organic and inorganic pollutants (Briggs and Staack, 2015; He et al., 2014; Praveen et al., 2013; Tahergorabi et al., 2012; Wang et al., 2016; Yu et al., 2009; Zhang et al., 2015). This technology utilizes electron accelerators to generate highly energetic (10 MeV) electrons from regular electricity. These electrons create both oxidative and reductive species via the radiolysis of water molecules which then further react with contaminants (Miller, 2005). The energetic electrons can also cause direct damage to inorganic and organic molecules.

Though eBeam technology is used in commercial medical device sterilization, food processing, phytosanitary applications, and wire and cable crosslinking industries, commercial adoption of the technology in the water industry is at its infancy (Gaspar, 2017). There have been a number of reports documenting the value of cobalt-60 (another ionizing radiation technology) based gamma irradiation technology for cyanobacteria inactivation and cyanotoxin degradation, but there are very few studies that have evaluated the use of eBeam technology for this purpose (Folcik and Pillai, 2020). Due to eBeam’s current lack of adoption, capital and operating costs are still being refined. However, in comparison to conventional and AOP technologies, eBeam’s ability to degrade numerous types of pollutants and organisms as well as its commercial electricity source make eBeam competitive economically (Meeroff et al., 2020; Pillai and Staack, 2020).

We have previously shown that when microbial cells are exposed to lethal doses of eBeam, the cells are inactivated and lose their ability to multiply. However, these inactivated cells can still be metabolically active for specific periods of time post eBeam exposure (Bhatia and Pillai, 2019; Jesudhasan et al., 2015; Rocha et al., 2019). We refer to these eBeam inactivated cells as Metabolically Active yet Non-Culturable (MAyNC).

The underlying hypothesis for this study was that when M. aeruginosa cells are exposed to lethal eBeam doses, the cyanobacterial cells would be unable to further synthesize microcystin. However, we also postulated that both intra- and extra-cellular microcystin would be degraded after eBeam exposure. Therefore, the specific research questions we pursued were a) to identify the eBeam dose that would be able to achieve inactivation of M. aeruginosa cells and degradation of extra-cellular MC-LR, b) determine the structural integrity of the eBeam-inactivated cells post eBeam exposure, and c) determine whether the intra-cellular MC-LR would be degraded with eBeam exposure.

## 2. Materials and methods

### 2.1. Laboratory propagation of M. aeruginosa

*M. aeruginosa* (LB 2385, UTEX Culture Collection of Algae, origin: Little Rideau Lake, Ontario, Canada) were cultured in a modified Bold 3N medium (without soil-water extract) under a 12/12 day/night cycle at ~20 °C on an orbital shaker at ~100 rpm. The cultures were also maintained on Bold 3N agar plates under identical light and temperature conditions. The cell titers were determined using chlorophyll absorbance (680 nm) read on a Synergy H1 Hybrid Multi-Mode Microplate Reader (Biotek, Winooski, VT) using Gen5 Microplate Reader and Imager software. An initially prepared standard curve was used for quantification. Cell titers were also determined microscopically just prior to experiments and throughout using a hemocytometer.

### 2.2. Quantification of Microcystin-LR

Pure microcystin-LR (purity ≥ 95%) was obtained commercially (Cayman Chemical, Ann Arbor, MI). Microcystin-LR concentrations in experimental samples were analyzed analytically at the Integrated Metabolomic Analysis Core (IMAC) at Texas A&M University. Supernatant samples were filtered through a 0.2 μm syringe filter and subjected to further methanol extraction. Cyanobacterial cell pellet samples were weighed (for wet weight normalization) and extracted using a methanol:chloroform:water based extraction method. Briefly, 800 μl ice cold methanol:chloroform (1:1, v:v) was added to cyanobacterial cell samples in a Precellys bead based lysis tube (Bertin, Rockville, MD). Samples were extracted on a Precellys 24 (Bertin) tissue homogenizer for 30 s at a speed of 6000. The supernatant was then collected and samples were homogenized a second time with 800 μl ice-cold methanol:chloroform. Following, 600 μl of ice-cold water was added to the combined extract, vortexed for 30 s, and centrifuged for 10 min at 4000 rpm at 4 ᵒC. The upper aqueous layer was filtered through a 0.2 μm nylon filter (Merck Millipore, Burlington, MA). 500 μl of the filtrate was then purified using a 3 kDa cutoff column (Thermo Scientific, Waltham, MA) and flow through collected for analysis.

Untargeted liquid chromatography high resolution accurate mass spectrometry (LC-HRAM) analysis was performed on a Q Exactive Plus orbitrap mass spectrometer (Thermo Scientific) coupled to a binary pump HPLC (UltiMate 3000, Thermo Scientific). Full MS spectra were obtained at 70,000 resolution (200 m/z) with a scan range of 100–1500 m/z. Full MS followed by ddMS2 scans were obtained at 35,000 resolution (MS1) and 17,500 resolution (MS2) with a 1.5 m/z isolation window and a stepped NCE (20, 40, 60). Samples were maintained at 4 °C before injection. The injection volume was 10 μl. Chromatographic separation was achieved on a Synergi Fusion 4 μm, 150 mm × 2 mm reverse phase column (Phenomenex, Torrance, CA) maintained at 30 °C using a solvent gradient method. Solvent A was water (0.1% formic acid). Solvent B was methanol (0.1% formic acid). The gradient method used was 0–5 min (10% B to 40% B), 5–7 min (40% B to 95% B), 7–9 min (95% B), 9–9.1 min (95% B to 10% B), 9.1–13 min (10% B). The flow rate was 0.4 ml min^−1^. Sample acquisition was performed with Xcalibur (Thermo Scientific). Data analysis was performed with Compound Discoverer 3.1 (Thermo Scientific).

### 2.3. Electron beam irradiation

The eBeam treatments were performed at Texas A&M University’s National Center for Electron Beam Research in College Station, TX. A high energy (10 MeV), 15 kW pulsed S-band linear accelerator was used (dose rate 3 kGy/s). Industry standard alanine (L-α-alanine) dosimeters and EPR based spectroscopy using the Bruker e-scan reader (Billerica, MA) were used to confirm delivered dose. Preliminary dose-mapping studies were performed on vials used for irradiation to confirm a dose uniformity of one. Studies involving pure microcystin were preformed using 2 ml glass screw-thread vials (VWR International, Radnor, PA). Larger 30 ml glass round-bottom screw cap culture tubes (Corning Inc., Corning, NY) were used for irradiation studies involving cyanobacterial cultures.

### 2.4. Response of M. aeruginosa cells to varying eBeam doses

High titers of *M. aeruginosa* cells (approximately 9 × 10^6^ cells/ml) were irradiated at target doses of 0, 0.6, 2, and 5 kGy. Actual doses received were 0.6, 2.1, and 4.9 kGy. Control and eBeam-treated cultures were then incubated for 14 days in 23 ml of fresh Bold 3N media in 125 ml Erlenmeyer flasks. Every 24 h, 1 ml aliquots were removed and cell concentrations were determined using the absorbance methods described above.

### 2.5. Microscopic examination of structural integrity

*M. aeruginosa* cells that received a lethal 5 kGy dose were observed microscopically using brightfield and fluorescence microscopy using a FITC filter. An Olympus BX50 microscope (Olympus, Shinjuku City, Tokyo, Japan) was used for this purpose. Images were captured using an Olympus Q color 3 camera and Qcapture pro 7 software (Teledyne QImaging, Surrey, British Colombia, Canada).

### 2.6. Stability and residual toxicity of microcystin-LR exposed to varying eBeam doses

Commercially purchased microcystin-LR was used in these studies. MC-LR was suspended in 2 ml of deionized water (0.5 mg/L) in 2 ml glass vials. This starting concentration of MC-LR was chosen to be a magnitude greater than the center of the standard curve of the EPA preferred ADDA-specific ELISA kit (Eurofins Abraxis inc., Warminster, PA) to allow for analytical detection. The ELISA kit standard curve ranged from 0.15 to 5.0 μg/L. These MC-LR samples were initially exposed to target eBeam doses of 5, 15, 25, 35, and 50 kGy. Following no detection of MC-LR, samples were then exposed to target doses of 0, 0.3, 0.4, 0.65, 2, and 5 kGy. Actual doses received were 0.29, 0.39, 0.64, 2.1, and 5.1 kGy. Quantification of MC-LR after irradiation was determined biologically using the EPA preferred ADDA-specific ELISA kit (Eurofins Abraxis) as well as analytically via LC-HRAM as described previously. This ELISA test kit follows guidelines set forth in the EPA method 546 for determination of total microcystins and nodularins in drinking water (Zaffiro et al., 2016). A protein phosphatase 2A inhibition kit (Eurofins Abraxis) was used as a basis for determining toxicity. The PP2A inhibition kit standard curve ranged from 0.25 to 2.5 μg/L. The underlying principle of this assay is that samples containing MC-LR will inhibit the PP2A enzyme proportionally to the amount contained in the sample. Normally, PP2A is able to hydrolyze a substrate that is detectable at 405 nm. MC-LR present in solution will inhibit the PP2A enzyme and prevent hydrolysis of the substrate.

### 2.7. Potential of microcystin-LR release from eBeam-exposed M. aeruginosa cells

Experiments were performed to determine whether lethally eBeam irradiated *M. aeruginosa* were capable of releasing MC-LR into the surrounding environment. For these experiments, turbid *M. aeruginosa* cultures (~10^7^ cells/ml) were exposed to target dose of 5 kGy which was determined earlier to achieve complete inactivation. Actual dose received was 5.4 kGy. The control and eBeam treated samples were incubated following treatment for 48 h. At periodic time intervals (0, 16, 24, and 48 h) 1 ml aliquots of culture were removed and centrifuged (5 min; 5000×*g*) to separate the cell pellets from the culture supernatant. The supernatant and the cell pellets were independently analyzed for the presence and concentrations of microcystin-LR according to methods described above. To preclude any possibility of cyanobacterial cells in the supernatant samples, the supernatant samples were syringe-filtered (0.2 μm) prior to methanol extraction for microcystin determination. The cell pellets were also weighed and extracted for microcystin determination.

### 2.8. Data analysis

All *M. aeruginosa* experiments were performed using biological triplicate samples. Additionally, the colormetric assays also included technical (n = 3) replicates. The data was statistically analyzed and visualized using commercially available GraphPad (GraphPad Software, San Diego, CA). Shapiro-Wilks tests and qq-plots were used to verify normality of data. According to these results, a one-way ANOVA was used followed by the Dunnett’s multiple comparisons test. The tests were performed with a significance of 95% (p < 0.05).

## 3. Results and discussion

### 3.1. Cyanobacterial inactivation

**Fig. 1.**
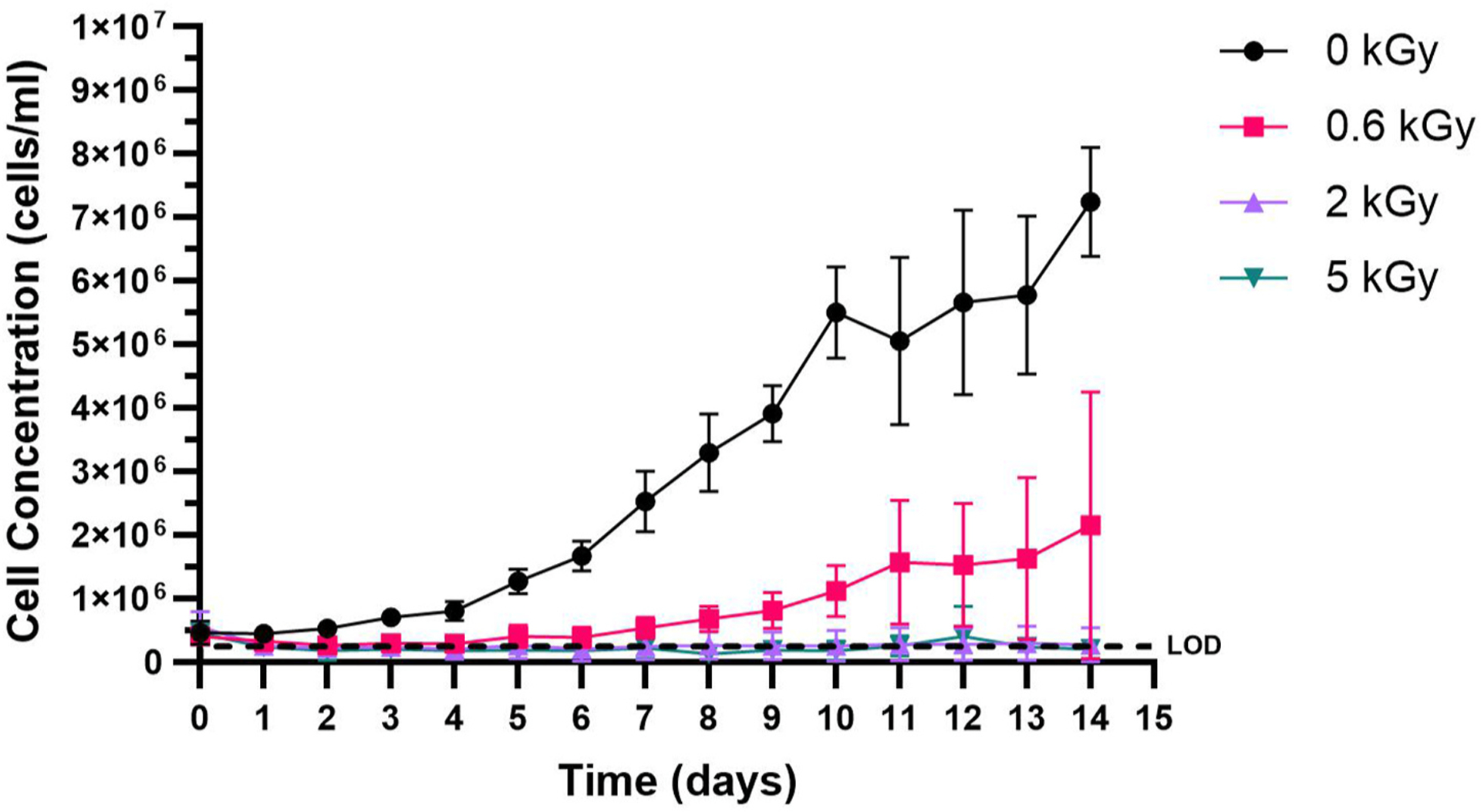
Response of *M. aeruginosa* cells to 0, 0.6, 2.1, and 4.9 kGy eBeam irradiation doses over 14 days. (Error bars represented as standard deviation, Limit of detection = 2.5 × 10^5^ cells/ml).

We first began by investigating the response of *M. aeruginosa* cells to electron beam irradiation. Fig. 1 shows the response of *M. aeruginosa* cells in liquid suspensions when exposed to varying eBeam doses which were then monitored over the course of 14 days using chlorophyll absorbance. The *M. aeruginosa* cells exposed to 2.1 and 4.9 kGy doses resulted in no cell multiplication over the 14-day monitoring period. Data values can be found in Supplemental Table 1. This suggests that *M. aeruginosa* cells (at 10^5^ cells/mL) are sensitive to eBeam irradiation doses even as low as 2 kGy and that doses >2 kGy are lethal doses. When exposed to 0.6 kGy, viable cells remained in the population which multiplied during the 14-day incubation period. However, the growth rate of the 0.6 kGy treated cultures were lower than that of the 0 kGy untreated control and did not show an increase in chlorophyll absorbance until 5 days of incubation following irradiation treatment. Therefore, there was sub-lethal injury at 0.6 kGy as evidenced by the reduced growth rate.

**Fig. 2.**
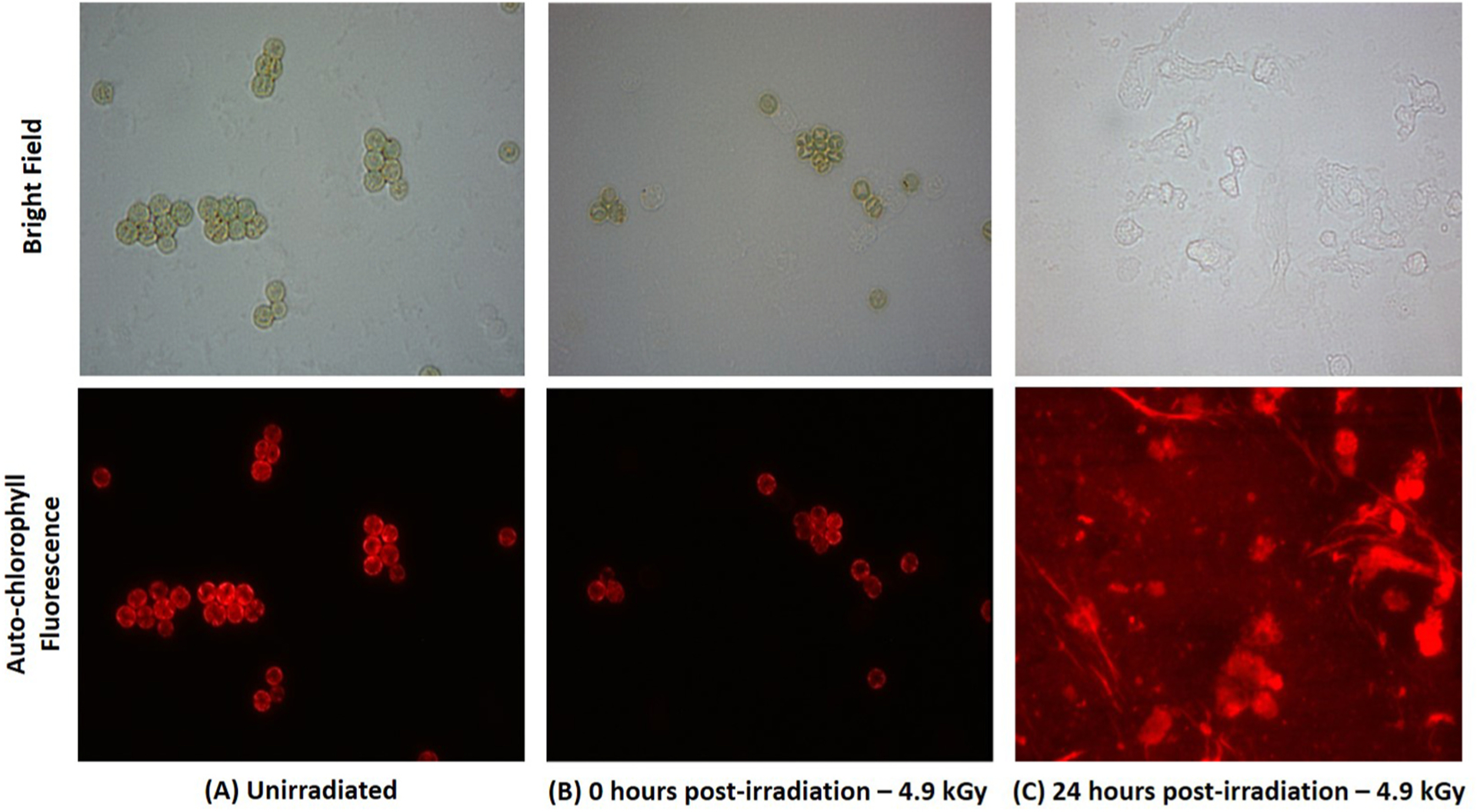
Brightfield and auto-chlorophyll fluorescence microscopy images of *M. aeruginosa* cells. A) Unirradiated cells. Cells appear normal and intact; B) Cells immediately after irradiation at 4.9 kGy. Cells are still structurally intact but have slight discoloring in the center of the cells indicating possible internal or membrane damage; C) Cells 24 h after irradiation at 4.9 kGy. Cells have completely lysed and free chlorophyll is fluorescing on the slide.

Cultures irradiated at 0.6, 2.1 and 4.9 kGy also all exhibited a visually detectable color change over time (Supplemental Figure 1). This color change implied a decline of chlorophyll pigments indicative of cell degradation (Zheng et al., 2012; Zhou et al., 2011). To investigate this further, brightfield and fluorescent microscopic images were taken of the *M. aeruginosa* cells when exposed to the 4.9 kGy dose (Fig. 2). The 4.9 kGy dosed cells were chosen for imaging because this was determined to be a lethal dose. Immediately after irradiation, *M. aeruginosa* cells still appeared structurally intact, however, there was slight discoloring in the centers of the cells indicating some internal or membrane damage. Cells were then imaged following 24 h of incubation after eBeam exposure at 4.9 kGy. After 24 h, the cyanobacterial cells appear to undergo lysis implying loss of structural integrity. The red hue in the 24 h auto-fluorescent micrograph is indicative of free chlorophyll fluorescing on the slide due to cell lysis.

The observed sensitivity of prokaryotic cells to eBeam doses is not surprising and has been reported extensively. This is the basis for the adoption of ionizing radiation, and eBeam in particular, for commercial pasteurization and sterilization applications in the food and medical devices industries (Lung et al., 2015; Pillai and Shayanfar, 2018; Silindir and Özer, 2009). However, the cell lysis seen in the irradiated cyanobacterial cells was surprising because other prokaryotes, such as *Salmonella enteriditis*, do not exhibit a decline in cell turbidity over time post-ionizing irradiation exposure (Jesudhasan et al., 2015). Only one other study identified completed by Agarwal et al. (2008) observed similar results in *Anacystis nidulans* exposed to gamma irradiation (Co^60^) (Agarwal et al., 2008). They observed significant alterations to the cyanobacterial cell ultrastructure and thylakoids following irradiation and 24 h of light exposure which they attributed to possible increases in glycogen deposits. Previous studies suggest that structural damage to photosynthetic machinery promotes cell death in photosynthetic organisms (Agarwal et al., 2008; Kovács and Keresztes, 2002; Strydom et al., 1991). Due to the necessity of photosystem function for cyanobacterial cell survival, it is possible that a similar mechanism is responsible for the unexpected cell lysis of eBeam exposed *M. aeruginosa* cells.

### 3.2. Stability and residual toxicity of microcystin-LR

**Fig. 3.**
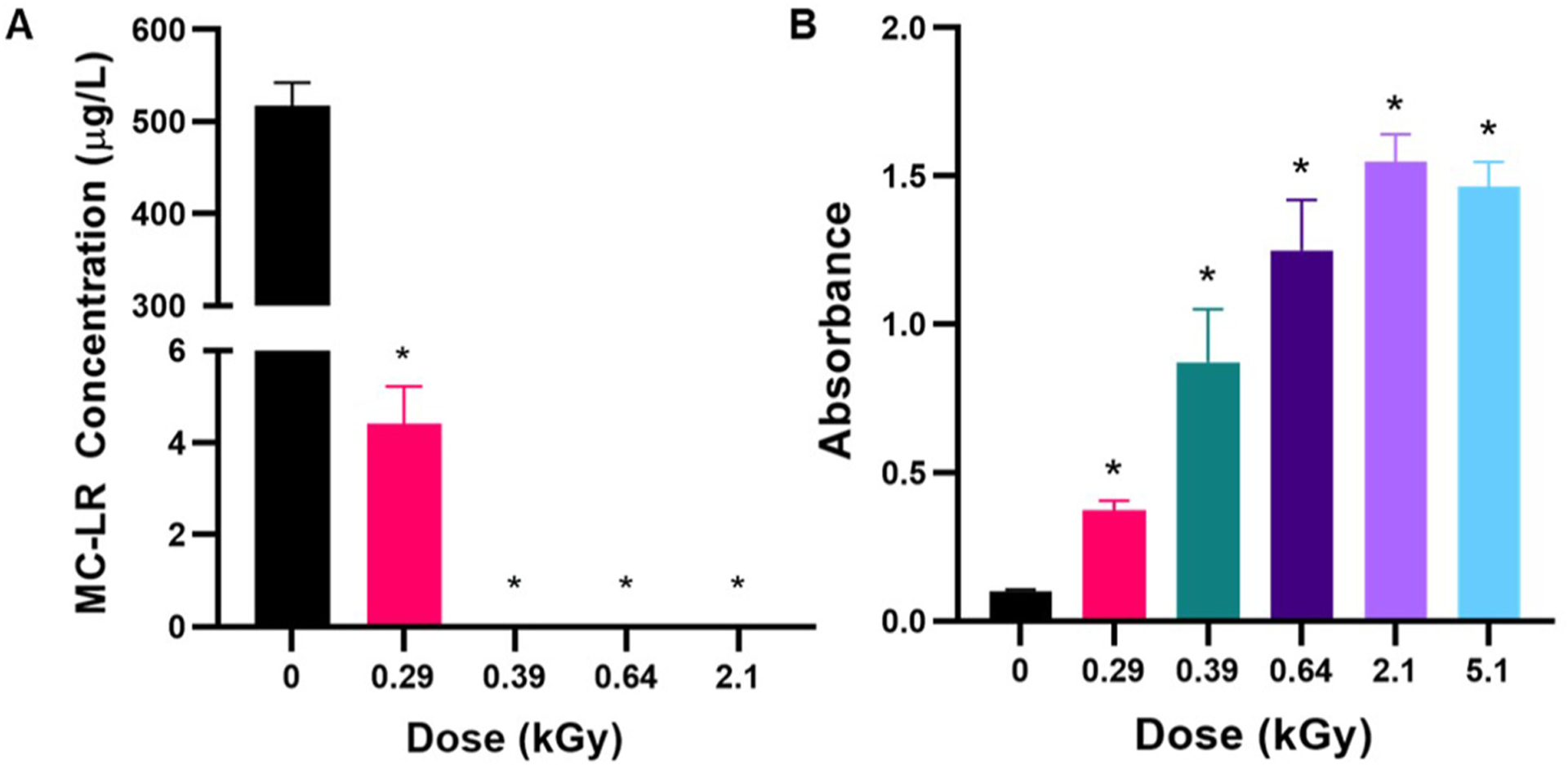
Degradation of MC-LR after 0, 0.29, 0.39, 0.64, 2.1, and 5.1 kGy eBeam irradiation treatment. A) Analytical determination of remaining MC-LR using LC-HRAM after eBeam treatment. B) MC-LR remaining after eBeam irradiation as determined by EPA method 546 ADDA-specific ELISA. Increasing absorbance corresponds to a decrease in binding of MC-LR to the detection antibody. (p ≤ 0.05; error bars represent standard deviation).

Following the investigation of the cellular effects of eBeam irradiation treatment on *M. aeruginosa*, we studied the effects of eBeam doses on the toxin, MC-LR. The response of pure MC-LR (0.5 mg/L) suspended in laboratory grade distilled water to 0, 0.29, 0.39, 0.64, 2.1 and 5.1 kGy is shown in Fig. 3. During preliminary studies, we had exposed 0.5 mM MC-LR to relatively high doses (between 5 kGy and 50 kGy) and attempted to detect the presence of the toxin molecule using LC-HRAM. However, all these doses resulted in the MC-LR concentrations dropping below the quantification limits (data not shown).

Fig. 3A depicts resulting MC-LR concentrations determined analytically using LC-HRAM. Samples irradiated at 0.29 kGy (290 Gy) resulted in an 85% reduction of the parent MC-LR concentration. At doses exceeding 0.29 kGy, MC-LR concentrations were below the limit of quantification (1 ng/L). Fig. 3B shows the remaining MC-LR following eBeam irradiation as determined biologically using the ADDA-specific ELISA kit (Eurofins Abraxis) (Zaffiro et al., 2016). Similarly to analytical identification, all eBeam treatment doses resulted in a significant decrease in binding within the assay suggesting degradation of the MC-LR parent molecule after eBeam irradiation treatment. These results suggest that minimal eBeam doses may be sufficient to breakdown extracellular MC-LR molecules in water samples.

**Fig. 4.**
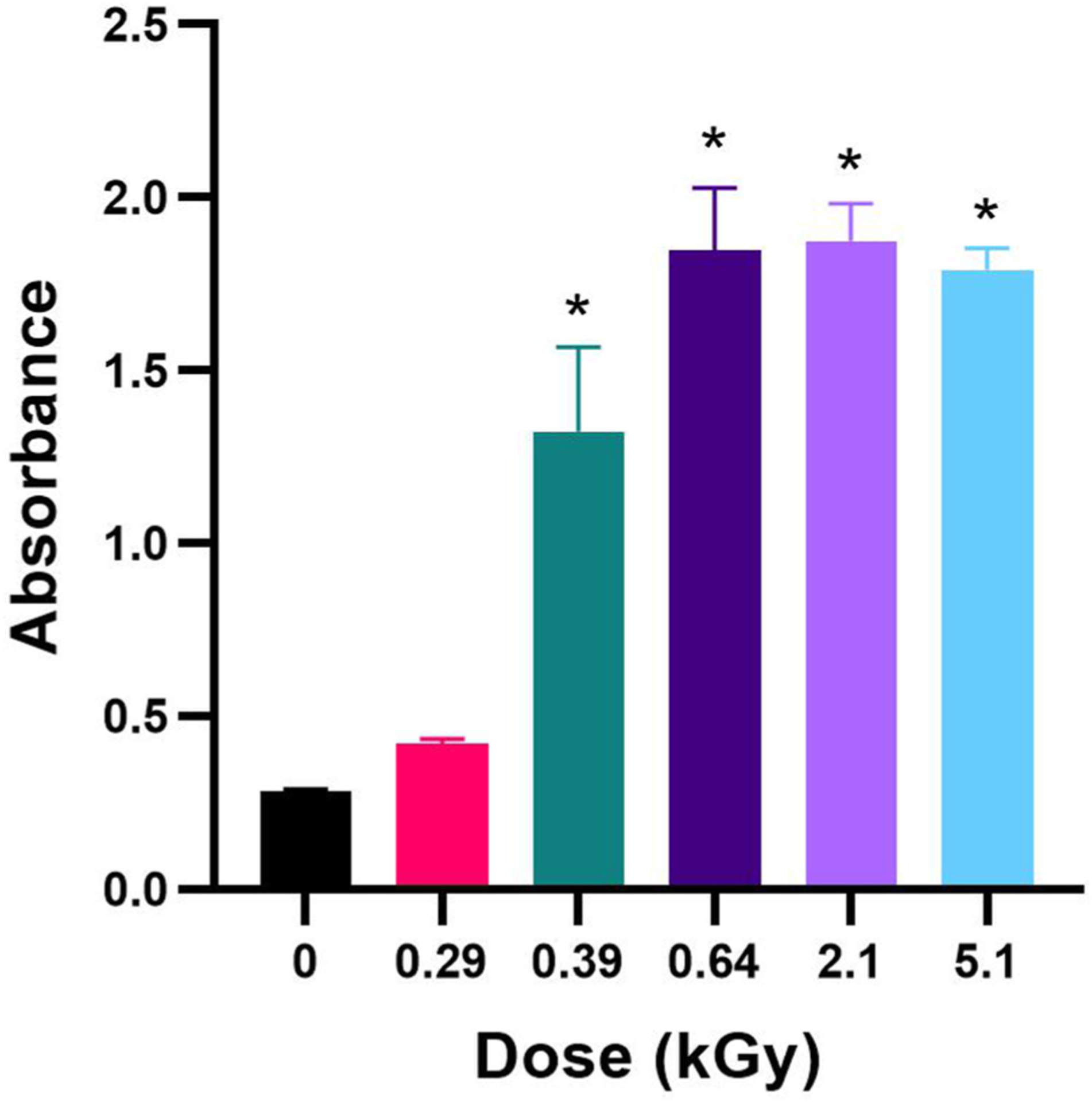
Protein phosphatase 2A inhibition assay toxicity of MC-LR after 0, 0.29, 0.39, 0.64, 2.1, and 5.1 kGy eBeam irradiation treatment as determined using a protein phosphatase 2A inhibition assay. (p ≤ 0.05; error bars represent standard deviation).

We then investigated the ability of MC-LR irradiation degradation products to exhibit toxicity using a colorimetric PP2A inhibition bioassay as previously described. We observed an increased absorbance corresponding to a decrease in PP2A inhibition at all doses (Fig. 4). This suggests that even at doses as low as 0.39 kGy, there is a significant reduction in toxicity. Samples treated with doses 0.64 kGy or greater resulted in similar levels of PP2A function. We were unable to use this assay quantitatively due to the MC-LR concentrations of irradiated samples falling below the kit’s standard curve concentrations. Nevertheless, these results indicate a significant reduction in PP2A inhibition in irradiated samples.

A portion of toxicity caused by MC-LR is associated with the binding and inhibition of protein phosphatases (Campos and Vasconcelos, 2010; Valério et al., 2010). This binding is thought to be largely due to the ADDA moiety of MC-LR molecule binding irreversibly to the enzyme (Craig et al., 1996). The gradual reduction of toxicity observed between 0.29 kGy and the 0.64 kGy treated MC-LR suggests that eBeam irradiation is resulting in possible structural damage to the ADDA moiety. Studies to understand the structure of the degradation products are currently underway.

Studies show that eBeam irradiation does cause both direct and indirect damage to biomolecules such as DNA and proteins (Desouky et al., 2015; Tahergorabi et al., 2012). It is understood that there is an inverse relationship between ionizing radiation doses required for degradation and the molecular weight of the target molecule (Miller, 2005; Osborne et al., 2000). Due to proteins being much smaller in size than DNA, our MC-LR degradation results were surprising. At present, little research has been done to identify the degradation products of MC-LR resulting from ionizing radiation treatment. Of these, a study completed by Song et al. (2009) identified two main possible reaction pathways of MC-LR degradation by gamma irradiation (Song et al., 2009). The first suggested pathway was the hydroxylation of the aromatic ring of the ADDA moiety, and the second was hydroxyl attack at the diene bond also on the ADDA group. However, in their studies, the solution was saturated with oxygen creating a more oxidative environment with the conversion of hydrated electrons to super oxide radical. Also, as mentioned above, there are fundamental differences in the form of ionizing radiation used. Further, it is unclear whether these degradation products of MC-LR will lack toxicity. The experiments that we have performed do not permit us to postulate whether the damage to the ADDA molecule is due to direct or indirect damage specifically. Studies are ongoing to determine residual toxicity of these degradation products in mammalian cells.

In comparison with our previous data on cyanobacterial cell response to eBeam irradiation, much less dose is required for the degradation of the MC-LR toxin. However, although Fig. 4 suggests doses >290 Gy are enough to breakdown MC-LR below quantification limits, it appears that the dose still leaves residual toxicity that is detectable by the PP2A inhibition bioassay (Fig. 4). This result emphasizes the higher sensitivity of bioassays compared to analytical assays (Humpage et al., 2010; Steinmann et al., 2011). Moreover, this result suggests that water utilities should include toxicity bioassays to determine residual toxicity rather than just relying on analytical determinations.

It is also important to note that these results utilize laboratory grade distilled water and cannot be extrapolated directly to the minimum dose required to achieve complete breakdown on 0.5 mM MC-LR concentration in drinking water supplies. This is because drinking water sample parameters can vary, primarily in terms of pH and alkalinity (Santana et al., 2014; Teixeira and Rosa, 2007; Wang and Chu, 2016). Nevertheless, the results suggest that at the pH of the laboratory distilled water (~7), low eBeam was sufficient to achieve significant breakdown. This result also suggests that the mechanism of breakdown is primarily hydroxyl radical mediated rather than the solvated electrons. To confirm the exact mode of MC-LR breakdown, the sequential use of radical scavengers needs to be employed (Ma et al., 2017).

### 3.3. Potential of microcystin-LR release from eBeam-exposed M. aeruginosa cells

It is recognized that eBeam inactivation of microorganisms results in the cells entering a MAyNC state (Bhatia and Pillai, 2019; Jesudhasan et al., 2015; Rocha et al., 2019). In order to understand the ability of eBeam irradiation to mitigate both cyanobacteria and cyanotoxins in water, it is also important to understand if *M. aeruginosa* is capable of retaining its metabolic activity. As noted above, it was observed that there is a delay in cell lysis following eBeam irradiation exposure. Therefore, we sought to investigate if MC-LR was present in cell culture following treatment. This was done by monitoring cultures post-irradiation over a 48-h time period and separating intra-cellular MC-LR from extra-cellular MC-LR for analysis.

**Fig. 5.**
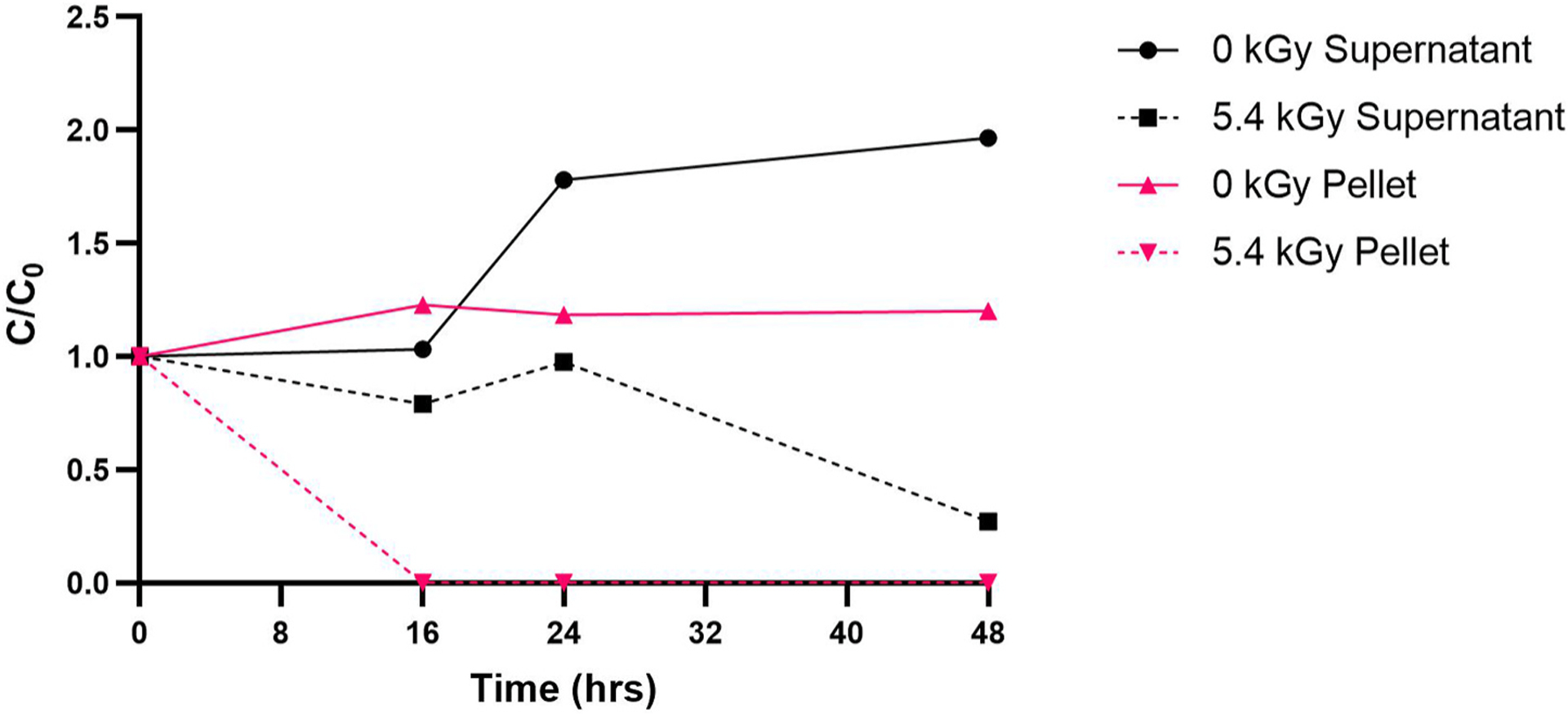
Time course study of intracellular MC-LR (pellet) and extracellular MC-LR (supernatant) at 0 and 5.4 kGy eBeam treatment.

Fig. 5 shows the intra-cellular and extra-cellular concentrations of MC-LR in *M. aeruginosa* cells after exposure to a lethal dose of 5.4 kGy and incubation in growth permissive Bold 3N medium. The intra-cellular and extra-cellular concentrations were based on the MC-LR concentrations in cell pellet and supernatant fractions, respectively. The MC-LR concentration in the untreated (0 kGy) cell pellet remained relatively constant over the 48 h. The MC-LR concentration in the untreated supernatant increased ~97% over 48 h due to normal cell turnover. In the lethally exposed (5.4 kGy) cell pellets, concentrations of MC-LR dropped below the limit of quantification at 16 h and remained below quantification limits up to 48 h. This drop in intra-cellular MC-LR was assumed to be indicative of cell lysis. On the other hand, the MC-LR concentration in the supernatant of cells exposed to lethal eBeam doses showed a gradual decline over 48 h but was not eliminated.

The presence of MC-LR in lethally exposed cell cultures suggests the possibility that *M. aeruginosa* is still metabolically active prior to cell death. During this time, synthesis of MC-LR may continue. The precise reasons why *M. aeruginosa* cells produce MC-LR and how these toxin molecules are transported out the of cells is still unknown. It is also unclear whether *M. aeruginosa* has the ability to actively transport MC-LR out of the cell or if MC-LR release is reliant on cell lysis (Pearson et al., 2004; Rohrlack and Hyenstrand, 2007). Fig. 2 indicates that when *M. aeruginosa* cells are exposed to a 4.9 kGy eBeam dose, the cells lyse within 24 h of eBeam exposure. Therefore, regardless of a mechanism of active transport, all remaining MC-LR is released into the surroundings. In Fig. 5, the increase of MC-LR concentrations in the supernatant of 5.4 kGy exposed cells (as compared to supernatant of unexposed cells) after lysis suggests that the majority of toxin release occurs during cell lysis. However, it is unknown if the presence of MC-LR after irradiation is a result of synthesis by the inactivated cells or remaining toxin that was not degraded in treatment. Further, there is a gradual decline of MC-LR in the 5.4 kGy eBeam treated supernatant over time. This could suggest that MC-LR produced by irradiated cells may be less stable than normal MC-LR or that changes to media composition after irradiation induce MC-LR degradation. This data then supports our hypotheses that *M. aeruginosa* cells are ‘inactivated’ and that extra-cellular microcystin is degraded. However, the presence of MC-LR in post-irradiated cell cultures contradicts our original predictions that no further synthesis would occur.

It is important to note that although resulting eBeam treatment concentrations of MC-LR in this study are lower than necessary for acute toxicity effects, chronic toxicity has been associated with prolonged MC-LR consumption. MC-LR is a potent acute toxin with an intraperitoneally administered LD_50_ of 25–150 μg/kg of body weight in mice (Fawell et al., 1994; Gupta, 1998). However, epidemiological studies have suggested increased risk of liver cancers following prolonged exposure to microcystin in drinking water (United States Environmental Protection Agency, 2006). A study completed by Zhou et al. (2002) also suggests there is an association between chronic microcystin exposure and colorectal cancer (Zhou et al., 2002). Therefore, understanding degradation and cellular effects are important for protecting the public’s clean water supply.

The finding that lethally inactivated *M. aeruginosa* cells could accumulate and release MC-LR into the surrounding medium prior to lysis is significant in terms of developing a drinking water treatment train. It is possible that with just a single lethal eBeam dose, the inactivated cells could still accumulate the toxin and release it into the surrounding water. Therefore, in addition to inactivating the cyanobacterial cells, a secondary eBeam exposure a few days post the initial exposure may be warranted. This is because residual MC-LR may remain in the water supply without proper down-stream treatment and pose further economic and public health ramifications. Alternatively, eBeam treatment may be deployed at the end of the drinking water treatment process following prior treatment that lyses the cyanobacterial cells. In this case, eBeam would be sufficient to remove free MC-LR from the water. However, a deeper understanding of *M. aeruginosa* cell’s metabolic activity post irradiation is necessary for development of effective treatment trains. Studies are on-going to understand the molecular mechanisms of how *M. aeruginosa* responds to eBeam irradiation treatment.

## 4. Conclusion

Ionizing radiation is utilized for a variety of applications due to its ability to reduce bioburden by damaging DNA and cell membranes in microorganisms. Often used for medical device sterilization or phytosanitation, ionizing radiation technologies present useful additions to water treatment processes to reduce both microbial loads as well as harmful toxins (Lung et al., 2015; Pillai and Shayanfar, 2018; Silindir and Özer, 2009; Wang et al., 2016). eBeam irradiation technology, in particular, is an electricity based and chemical free treatment strategy that could be effective at mitigating cyanoHABs and their toxins within the water treatment process.

In order for adequate treatment, it is imperative to understand the fundamental biology and chemistry of ionizing radiation on the various target microorganisms and pollutants. In summary, this study shows that eBeam irradiation can achieve significant reduction of MC-LR in water. Residual degradation products of MC-LR are unable to inhibit protein phosphatase activity suggesting an alleviation of toxicity. eBeam irradiation is also able to inactivate *M. aeruginosa* cells by inducing cell lysis. To our knowledge, this behavior has not been previously reported in cyanobacteria following eBeam irradiation. However, cell lysis occurs on a time delay after irradiation treatment. Additionally, *M. aeruginosa* cells enter a MAyNC state during this time and may have the ability to synthesize more microcystin prior to cell lysis.

While full scale eBeam technology platforms for environmental remediation and water treatment are still in their infancy, this study demonstrates the utility of eBeam technologies for cyanotoxin and cyanobacteria degradation. In comparison with conventional and AOP technologies, eBeam technology is able to affect both toxin and organisms in water. Further studies are necessary to understand cellular response in this time frame after treatment and before lysis. These preliminary studies were a primary screen for general cellular responses but represent a basis for further detailed research.

## Supplementary Material

Supplementary Table 1

Supplementary Figure
