# Supplementary material for: Response of Microcystis aeruginosa and Microcystin-LR to electron beam irradiation doses
Source: Radiat Phys Chem Oxf Engl 1993. Author manuscript; Available in PMC 2021 Sep 1. (PMC8143040; doi:10.1016/j.radphyschem.2021.109534)
Supplement: Supplementary Table 1 [file NIHMS1703117-supplement-Supplementary_Table_1.docx]

| **Day** | **0 kGy** | **0.6 kGy** | **2.1 kGy** | **4.9 kGy** |
| --- | --- | --- | --- | --- |
| **0** | 4.6x10^5^ ± 8.1x10^4^ | 4.1x10^5^ ± 0.0 | 5.5x10^5^ ± 1.4x10^5^ | 4.6x10^5^ ± 8.1x10^4^ |
| **1** | 4.5x10^5^ ± 1.2x10^4^ | 3.3x10^5^ ± 4.6x10^4^ | 2.6x10^5^ ± 1.2x10^4^ | <LOD |
| **2** | 5.3x10^5^ ± 2.3x10^4^ | 2.6x10^5^ ± 0.0 | <LOD | <LOD |
| **3** | 7.0x10^5^ ± 1.2x10^4^ | 3.0x10^5^ ± 1.2x10^4^ | <LOD | <LOD |
| **4** | 8.1x10^5^ ± 5.3x10^4^ | 2.9x10^5^ ± 2.0x10^4^ | <LOD | <LOD |
| **5** | 1.3x10^6^ ± 9.0x10^4^ | 4.0x10^5^ ± 8.0x10^4^ | 2.6x10^5^ ± 1.1x10^5^ | <LOD |
| **6** | 1.7x10^6^ ± 1.3x10^5^ | 3.9x10^5^ ± 5.8x10^4^ | <LOD | <LOD |
| **7** | 2.5x10^6^ ± 3.8x10^5^ | 5.4x10^5^ ± 7.0x10^4^ | <LOD | <LOD |
| **8** | 3.3x10^6^ ± 5.2x10^5^ | 6.8x10^5^ ± 1.0x10^5^ | 2.6x10^5^ ± 1.2x10^5^ | <LOD |
| **9** | 3.9x10^6^ ± 3.5x10^5^ | 8.2x10^5^ ± 1.9x10^5^ | 2.6x10^5^ ± 1.2x10^5^ | <LOD |
| **10** | 5.5x10^6^ ± 6.2x10^5^ | 1.1x10^6^ ± 3.0x10^5^ | 2.6x10^5^ ± 1.5x10^5^ | <LOD |
| **11** | 5.1x10^6^ ± 1.2x10^5^ | 1.6x10^6^ ± 8.8x10^5^ | 2.8x10^5^ ± 1.6x10^5^ | 2.6x10^5^ ± 6.1x10^4^ |
| **12** | 5.7x10^6^ ± 1.4x10^5^ | 1.5x10^6^ ± 8.7x10^5^ | 2.8x10^5^ ± 1.6x10^5^ | 4.0x10^5^ ± 3.8x10^5^ |
| **13** | 5.8x10^6^ ± 2.0x10^5^ | 1.6x10^6^ ± 1.2x10^6^ | 3.0x10^5^ ± 2.1x10^5^ | <LOD |
| **14** | 7.3x10^6^ ± 7.6x10^5^ | 2.2x10^6^ ± 2.0x10^6^ | 2.7x10^5^ ± 1.7x10^5^ | <LOD |

**Supplementary Table 1.** Response of *M. aeruginosa* cells to 0, 0.6, 2.1, and 4.9 kGy eBeam irradiation doses over 14 days. Cell concentrations were determined using chlorophyll absorbance at 680 nm using a microplate reader. Data is expressed as cells/ml ± standard deviation. (Limit of detection = 2.5x10^5^ cells/ml).
