# Supplementary figures and images for: Response of Microcystis aeruginosa and Microcystin-LR to electron beam irradiation doses
Source: Radiat Phys Chem Oxf Engl 1993. Author manuscript; Available in PMC 2021 Sep 1. (PMC8143040; doi:10.1016/j.radphyschem.2021.109534)

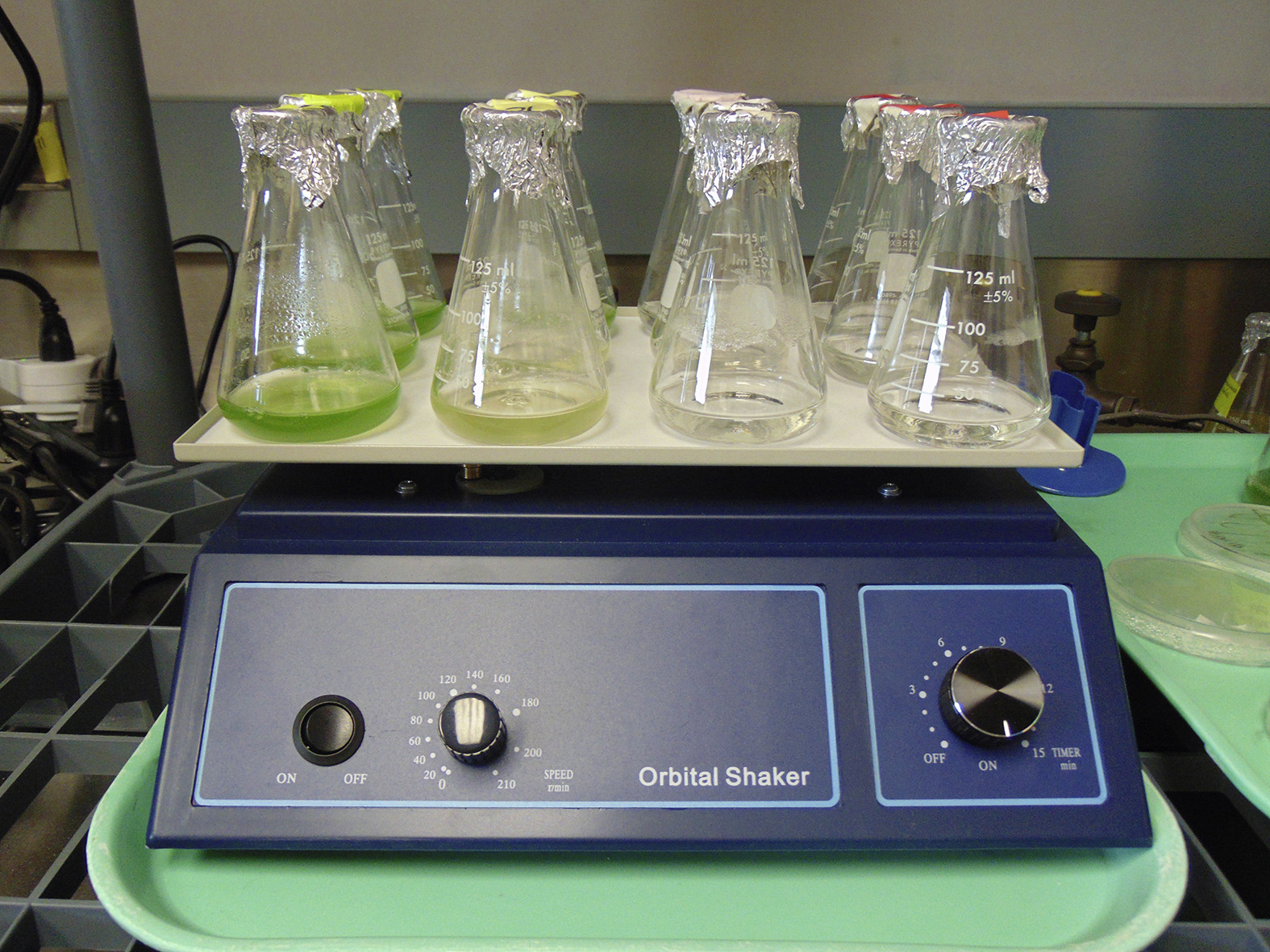

Supplement: Supplementary Figure [file NIHMS1703117-supplement-Supplementary_Figure.jpg]
